# On the emerging relationship between the stratospheric Quasi-Biennial oscillation and the Madden-Julian oscillation

**DOI:** 10.1038/s41598-019-40034-6

**Published:** 2019-02-27

**Authors:** P. Klotzbach, S. Abhik, H. H. Hendon, M. Bell, C. Lucas, A. G. Marshall, E. C. J. Oliver

**Affiliations:** 1 Department of Atmospheric Science, Colorado State University, Fort Collins, CO USA; 2 School of Earth, Atmosphere & Environment, Monash University, Clayton, Australia; 3 Bureau of Meteorology, Melbourne, Australia; 4 Bureau of Meteorology, Hobart, Australia; 5 Department of Oceanography, Dalhousie University, Halifax, Nova Scotia Canada

## Abstract

A strong relationship between the quasi-biennial oscillation (QBO) of equatorial stratospheric winds and the amplitude of the Madden-Julian oscillation (MJO) during the boreal winter has recently been uncovered using observational data from the mid-1970s to the present. When the QBO is in its easterly phase in the lower stratosphere, it favors stronger MJO activity during boreal winter, while the MJO tends to be weaker during the westerly phase of the QBO. Here we show using reconstructed indices of the MJO and QBO back to 1905 that the relationship between enhanced boreal winter MJO activity and the easterly phase of the QBO has only emerged since the early 1980s. The emergence of this relationship coincides with the recent cooling trend in the equatorial lower stratosphere and the warming trend in the equatorial upper troposphere, which appears to have sensitized MJO convective activity to QBO-induced changes in static stability near the tropopause. Climate change is thus suggested to have played a role in promoting coupling between the MJO and the QBO.

## Introduction

The Quasi-Biennial oscillation (QBO) is characterized by downward propagating alternating easterly and westerly winds in the equatorial lower stratosphere with a period of approximately 28 months^1^. Although the QBO is a stratospheric phenomenon, it has been linked to changes in many aspects of tropical convection, including variations of Indo-Pacific deep convection^2,3^ and near-tropopause cirrus^4,5^, variations in tropical cyclone frequency and tracks in the Atlantic^6,7^ and North Indian Oceans^8^, variations in subtropical jet streams and subtropical precipitation^9–13^, and variations in teleconnections associated with El Niño-Southern Oscillation^14,15^.

The Madden-Julian oscillation^16^ dominates tropospheric tropical subseasonal variability and is characterized by a large-scale (~10,000 km) region of enhanced deep convection, coupled to a zonal overturning circulation through the depth of the troposphere, which together propagate eastward along the equator with a local period of 30–70 days and an eastward phase speed of ~5 ms^−1^. The convective component of the MJO is most prominent across the warm waters of the equatorial Indian and western Pacific Oceans, while the atmospheric circulation disturbance extends across the entire tropics. Besides dominating subseasonal variability of rainfall and surface winds across the Indo-Pacific warm pool, the MJO drives variations in a wide variety of atmospheric phenomena including active and break cycles of the Indian and Australian summer monsoons^17,18^, tropical cyclones around the globe^19^, surface temperatures across North America^20–22^ and the North Atlantic Oscillation^23^, and so is an important source of subseasonal climate predictability^24^.

The amplitude of MJO activity during boreal winter, when the MJO is typically most active, has recently been shown to be enhanced when the QBO is in its easterly phase in the lower stratosphere (hereafter referred to as QBOE) and suppressed when it is in its westerly phase (hereafter referred to as QBOW)^25^. The year-to-year variation of MJO activity attributed to the QBO is much greater than from interannual variations of tropical sea surface temperatures^5,26^, which are typically the major source of interannual variations of tropical convective activity. Marshall *et al*.^27^ further showed that the boreal winter MJO is more predictable during QBOE, with a skill increase of ~1-week lead-time compared to during QBOW. This improvement in skill represents the largest known impact of any climate driver for MJO predictability.

The enhanced predictive skill for the MJO during QBOE is not simply due to a higher signal to noise ratio because of greater MJO activity. Rather, enhanced predictability derives from altered behavior of the MJO whereby its amplitude is more persistent during QBOE^27^. This enhanced persistence reflects more continuous propagation of the convective phase of the MJO from the Indian Ocean, across the Maritime Continent and into the western Pacific^28,29^. The implications of the relationship between the boreal winter MJO and the QBO are considerable because the QBO is readily predictable several months into the future^30^, consequently leading to the potential for improved and more confident prediction of weather and climate for portions of the globe where the MJO plays a significant role.

Although the observed QBO-MJO relationship is highly significant and strong (i.e., the QBO explains ~50% of the year-to-year variance of boreal winter MJO activity^5,25,27,28^), the relationship has been established using the relatively short record of data during the satellite era (post mid-1970s) when the global behavior of the MJO is well constrained by satellite observations of convection. Despite a plausible physical explanation having been suggested to account for increased MJO activity during QBOE (e.g., reduction in the stratification of temperature between the upper troposphere and stratosphere)^5,25,28,31^, the question is raised as to whether a similar relationship existed prior to the satellite era. This question is especially pertinent considering recent trends in temperature in the upper troposphere and lower stratosphere that have acted to destabilize the equatorial tropopause^32–34^, which could have possibly promoted the recent modulation of the MJO by the QBO. To address this question, we investigate the MJO-QBO relationship back to 1905 using reconstructions of the MJO and the QBO. Although the confidence in these reconstructions reduces prior to ~1958 when regular radiosonde observations first began on a global basis, the analysis here reveals that the relationship between the MJO and QBO appears to have emerged only since the early 1980s, when data quality is not an issue. Consequently, we also examine potential mechanisms to explain this recent emergence.

## Methods

During the “satellite era”, which refers here to the period beginning in 1974 when global satellite observations of tropical convective clouds, winds and temperatures became routinely available for direct monitoring of convection and for assimilation into global atmospheric reanalyses, the MJO can be monitored either solely by its convective signature^35^ or by its combined signal in winds and convection^36^. Similar relationships between boreal winter MJO activity and the QBO are obtained using either the convection-based or the convection-wind based indices of the MJO^5,25,28,31^. In this study we adopt the approach of Wheeler and Hendon^36^ to identify the MJO using a bivariate index developed from a combination of equatorial zonal winds and convection.

For the satellite era, we define the MJO using the Realtime Multivariate MJO (RMM) index derived by Wheeler and Hendon^36^, hereafter WH, which are formed using satellite-observed outgoing longwave radiation (OLR) and zonal winds at 200 and 850hPa from the National Centers for Environmental Prediction (NCEP)-National Center for Atmospheric Research (NCAR) Reanalysis^37^. In order to extend the RMM index back to 1958, we make use of zonal winds and OLR directly provided by the JRA-55 Reanalysis^38^. JRA-55 is the longest third-generation reanalysis that uses the full observing system. It uses an advanced 4DVar data assimilation scheme, a new variational bias correction for satellite data, and several additional observational data sources, which in comparison with previous versions has resulted in reduced biases in stratospheric temperature and greater temporal consistency of the temperature analyses. The JRA-55 commenced in 1958 when routine global radiosonde observations began.

The MJO index derived from JRA-55 was calculated by initially bandpass filtering OLR, and zonal winds at 200 and 850 hPa for periods between 2–120 days for the entire record. The period from 1958–2014 is used here, which approximates the data preparation technique used in WH (they removed the annual cycle and subtracted a 120-day mean). Then, following the procedure of WH, the zonal winds and OLR were equatorially averaged and normalized by the square root of the respective zonal mean variances provided by WH. These normalized fields were then projected onto the pair of Empirical Orthogonal Functions (EOFs) that were derived by WH using zonal winds from the NCEP-NCAR Reanalysis^37^ and OLR from the Advanced Very High Resolution Radiometer (AVHRR)^39^. The RMM indices derived from JRA-55 were then normalized by the square root of the eigenvalues provided by WH, which were derived using data from 1979–2001.

For the period of overlap of the WH and JRA RMM indices available in this study (1974–2014), the correlation of the daily individual RMM indices exceeds 0.9 (Table 1). This does not confirm the fidelity of the JRA index prior to the satellite period when the WH RMM indices are not available, but the favorable comparison during the period of overlap provides considerable confidence in its quality.

**Table 1:** Inter-correlation matrix for daily values of RMM1 (RMM2) during 1974–2014 derived from WH, JRA, and OT.

	WH	JRA
OT	0.83 (0.83)	0.82 (0.81)
WH		0.91 (0.92)

In order to extend the MJO indices before 1958, the WH RMM indices have also been reconstructed for 1905–2014 by using point “observations” of surface pressure across the tropics as predictors in a linear regression model^40^, hereafter referred to as the OT index. This approach exploits the strong signature of the MJO in surface pressure^16^. The surface pressure “observations” are taken from the 20^th^ Century Reanalysis Project^41^ (20CR), which is a global analysis that assimilates only surface pressure observations. The 20CR is based on an ensemble Kalman filter and so provides 56 equally probable realizations. We make use of the 56 realizations to produce a 56-member ensemble of the RMM indices, whose spread is indicative of observational uncertainty. The uncertainty of the reanalysis increases in earlier years due to a reduced number of surface pressure observations, but since 1958 the reanalysis has been shown to perform well, capturing both tropical and extratropical synoptic variability^41^. The regression to reconstruct the RMM was developed using dependent data for 1979–2008 so that the temporal properties of the reconstructed RMM indices are consistent with WH for this period. The analysis in this paper ends in 2014 as the 20CR ends in December 2014.

For the period of overlap of the OT index with the WH and JRA indices (1974–2014), the correlation of the daily individual RMM indices exceeds 0.8 (Table 1). The general validity of the RMM indices derived by OT for the period prior to the “satellite era” is demonstrated by the recovery of consistent relationships between the MJO with other independently observed climate variables over the course of the 20th century including the intraseasonal variation of tropical cloud coverage and sea surface height^40^, air temperature over Alaska^42^, Atlantic and global tropical cyclones^43,44^, the frequency of nor’easters near the northeast United States coast during boreal winter^45^, and heavy rainfall over northern Australia^46^.

In this study, we focus on the DJF mean MJO amplitude. We define the daily amplitude of the MJO following Yoo and Son^25^ and Marshall *et al*.^27^ as the square root of the sum of the two daily indices squared (|RMM | = (RMM1^2^ + RMM^2^)^1/2^. We then form the seasonal mean of |RMM| for each December-January-February season, labeling the year based upon January. For instance, 1959 is the average of December 1958, January 1959 and February 1959. We form these yearly amplitude time series from the three sets of RMM indices, which we hereafter refer to as the WH, JRA and OT indices.

**Figure 1:**
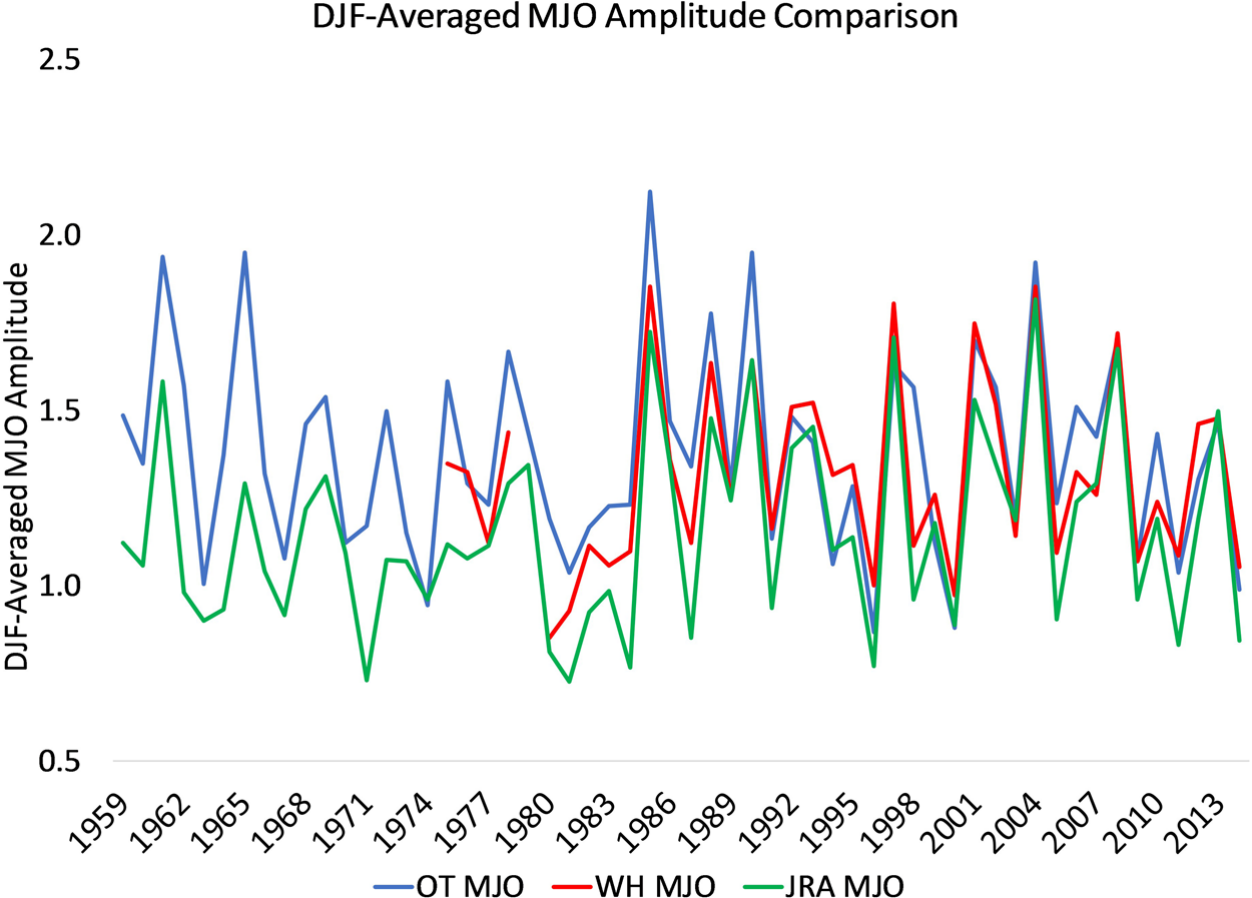
Time series of DJF-mean amplitude from WH, JRA, and OT. For the period 1979–2014 r(OT,JRA) = 0.85, r(OT,WH) = 0.86, and r(WH,JRA) = 0.95. For the period 1959–1978, r(OT,JRA) = 0.76.

The time series of the DJF mean amplitude of the MJO using the three different indices are displayed in Fig. 1 for the complete period of overlap (1959–2014). Very similar interannual variations are detected in all three indices. For the period 1979–2014, the correlation of the MJO amplitude from the three indices all exceed 0.85, with the correlation between WH and JRA exceeding 0.9 (r = 0.95). We also find close correspondence between the OT and JRA indices over the joint pre-satellite period of 1958–1978 (r = 0.76). The mean amplitude of the JRA index is slightly weaker than the WH index (1.19 versus 1.31 for DJF 1979–2014). This appears to stem from weaker OLR variability in the JRA reanalyses compared to AVHRR data^47^, which were used to derive the WH index. Our approach to derive the RMM from JRA is to use normalizations based on WH, so that the weaker OLR variability in JRA results in weaker MJO amplitude compared to WH. Note that the OT reconstruction constrained the RMM indices to have the observed (WH) amplitude during the dependent training period. For reference, the average OT index amplitude from 1906–1958 is 1.40.

The QBO is depicted using indices of equatorial zonal mean zonal wind at 50 hPa for two consecutive periods. For 1905–1953, when direct observations of the stratosphere are not numerous^1^, we use the reconstruction from Broennimann *et al*.^48^. This QBO index is reconstructed through regression using surface pressure observations that are coherent with the QBO. This time series is standardized using a 1911–1940 base period.

For the period post-1953, we use the station-based radiosonde QBO index from the Free University of Berlin (FUB). This dataset was created using three near-equatorial stations: Canton Island, Gan Island and Singapore. Additional information on the original data going into this dataset is available in Naujokat^49^. We also calculated a QBO index directly using monthly mean equatorially-averaged (5°S-5°N) zonal mean zonal wind data at 50 hPa from the JRA-55 Reanalysis^30^. For the post-1957 period the correlation of the JRA QBO index with the FUB index is 0.98 (Supplementary Fig. S1). The FUB timeseries is standardized using a 1981–2010 base period.

We define the easterly QBO phase when the December-February mean index values are negative, and the westerly phase when the mean index values are positive. This QBO definition is the same as used in Hamilton^50^. We also define strong QBO years when the index exceeds plus or minus 0.5 standard deviations, similar to the approach of Son *et al*.^5^, for data analysis displayed in the final four figures of the manuscript.

### Association of the MJO with the QBO 1905–2014

**Figure 2:**
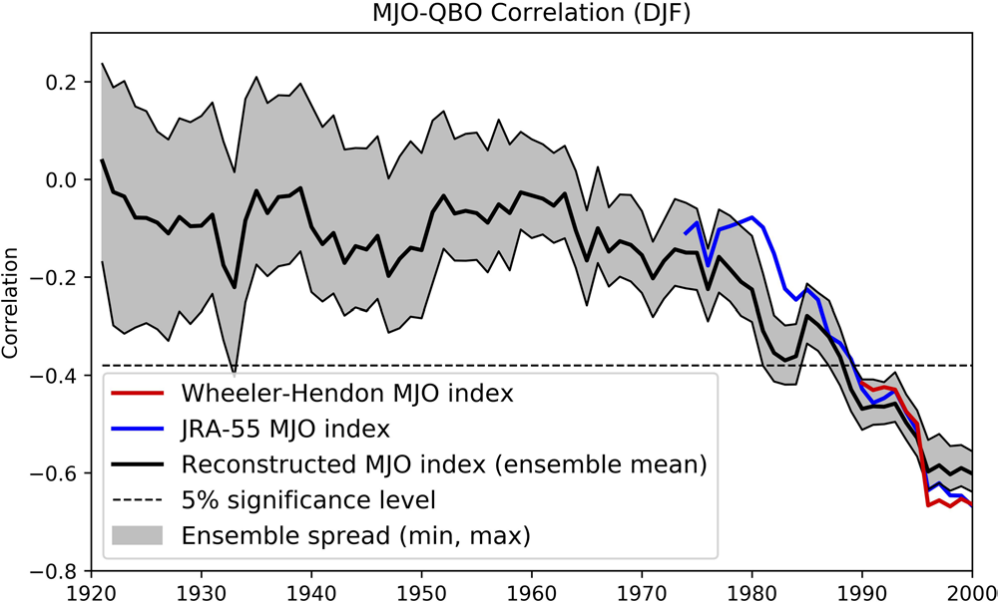
30-year running correlation between DJF-averaged MJO amplitude and the QBO index. Correlation values are shown using the Wheeler-Hendon MJO index (red line), the JRA-55 MJO index (blue line) and the long-term reconstructed MJO (OT) index (thick black solid line) with the ordinate on the x-axis given by the central year of the 30-year running window. Maximum and minimum 30-year running correlations for the reconstructed index are also displayed (thin black solid lines), calculated from the 56 members of the long-term MJO index ensemble. The dashed line represents the 5% statistical significance level.

We explore the boreal winter MJO-QBO relationship by computing the correlation of December-February mean MJO amplitude with the QBO index in a sliding 30-year window (Fig. 2). The right most plotted point in Fig. 2 is the correlation based on data for the most recent 30- year period 1985–2014 and produces the previously reported strong negative correlation between the QBO and MJO amplitude for this period^25^ (r = ~−0.7). The correlations computed using the WH and JRA indices for the 1985–2014 periods exhibit similar values and are slightly stronger than from using OT. Going backwards in time, the correlations using all three indices systematically weaken, with the correlation using OT and JRA indices displaying similar declines back to the 30-yr window centered on 1959–1988. Prior to this time, the correlation using OT fluctuates around ~−0.2 back to the beginning of the record. For all three indices, the statistically significant negative correlation (p = 0.05) emerges for the data window beginning with ~1970–2000. This finding of a systematically increasing negative correlation since ~1960 is relatively insensitive to the length of the sliding window used to calculate the correlation (Supplementary Fig. S2).

We additionally quantify uncertainty in the correlation based on OT by displaying the spread in the correlation derived from each of the 56 members from the 20CR (grey area about the black dashed curve in Fig. 2). The spread in the correlation increases backwards in time especially prior to 1959–1988. However, the weakening of the correlation prior to the late 1950s is greater than the increase in spread, thus providing more confidence that a significant relationship between the QBO and the MJO has only emerged since ~1980.

**Figure 3:**
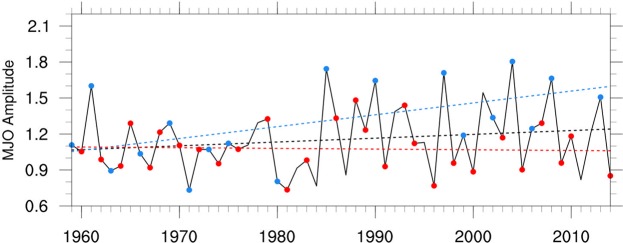
Seasonal (DJF) mean JRA index MJO amplitude and its trend (black line) based on all DJF seasons since 1959. Linear trends are also computed for QBOE years (blue line) and QBOW years (red line). Strong QBOE and QBOW years are indicated by blue and red dots, respectively.

A consistent explanation for the increasing correlation of the MJO amplitude with the QBO since ~1960 is an increasing trend of the MJO amplitude during QBOE, and possibly a decreasing trend in MJO amplitude during QBOW. The JRA MJO index indeed exhibits a statistically significant (p = 0.02) upward trend during QBOE along with a weaker and not significant (p = 0.2) downward trend during QBOW for 1959–2014 (Fig. 3). Similar trends are exhibited using the OT index (Supplementary Fig. S3).

Based on the JRA index, the MJO amplitude during east years has increased by about 2 standard deviations of the December-February-averaged MJO amplitude since 1959. These trends are reflected in the amplitude of the MJO during west and east years being approximately equal for the period 1959–1979, while during 1980–2014 the amplitude during east years is ~33% larger than during west years (~25% larger using the WH index for the 1980–2014 period). The increasing trend in MJO amplitude in QBOE may be a step increase in amplitude in the mid-1980s (Supplementary Fig. S4), but large year-to-year variations make it difficult to ascertain from a constant trend. Synthetic data for 60-year periods demonstrates that an upward trend of MJO amplitude during QBOE since 1959, which increases as observed by ~2 standard deviations over the period 1959–2014, can account for the strengthening of the negative correlation from ~−0.2 for the 1959–1988 period to ~−0.7 for 1985–2014 (Supplementary Fig. S5). A similar continual strengthening of the negative correlation over 60 years also emerges from the synthetic data if the MJO amplitude is assumed to make a step increase during QBOE after the first 30 years.

### Mechanism for the emerging relationship

**Figure 4:**
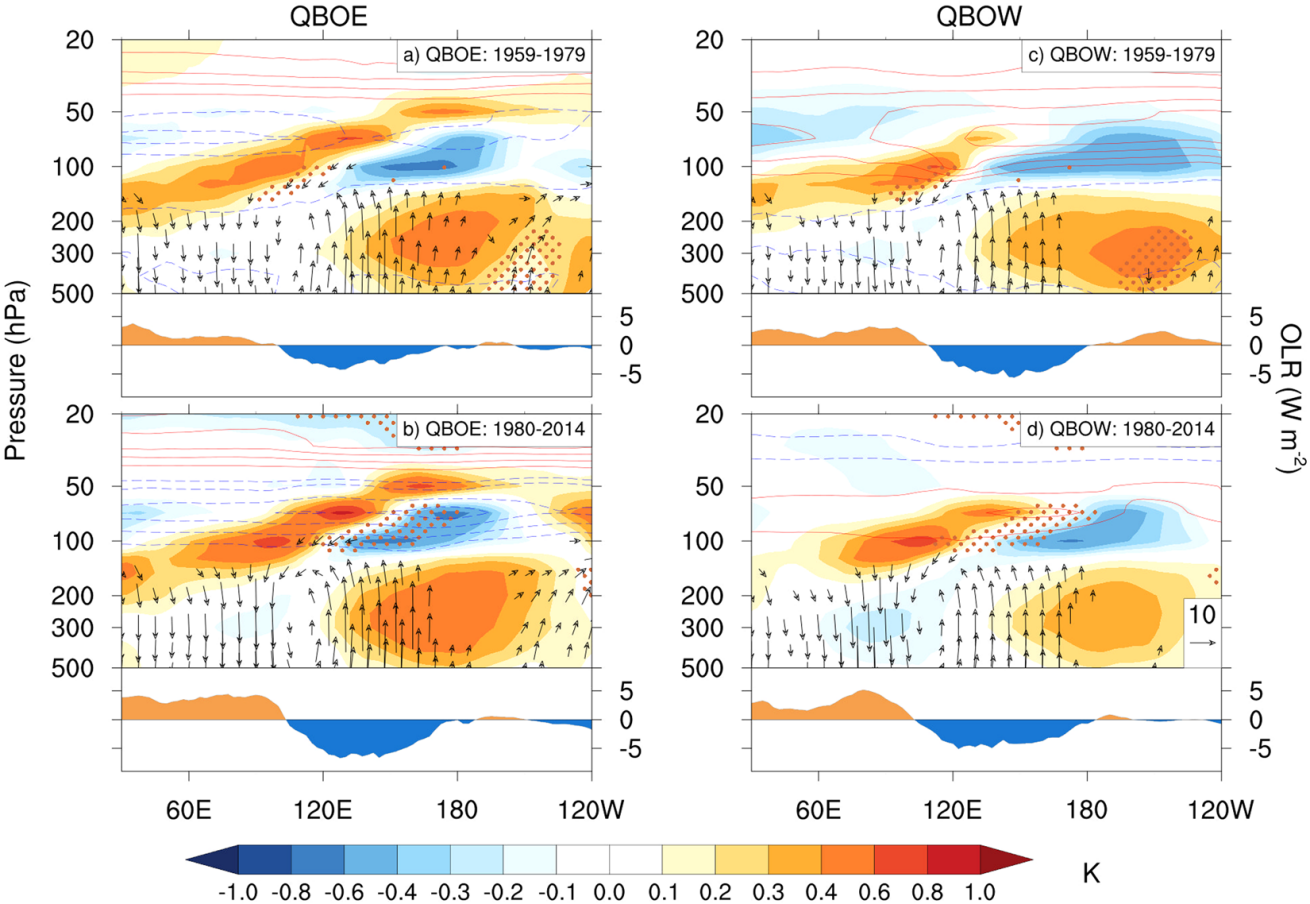
Regression of DJF equatorially averaged (10°S-10°N) JRA temperatures (shading), and zonal and vertical winds (vectors; scale in lower right panel) onto the JRA RMM1 + RMM2 time series for QBOE (left column) and QBOW (right column). Below each panel is the regression of JRA OLR onto the JRA RMM1 + RMM2 index. The top row is for the period 1959–1979 and the bottom row is 1980–2014. The mean temperature anomaly for QBOE and QBOW for each period relative to the climatology for 1980–2010 is contoured (contour interval 0.1°K). The vector scale indicates a 10 ms^−1^ zonal wind or 7.41 × 10^−3^ Pa s^−1^ vertical velocity anomaly. The vertical velocity has been multiplied by −1 so that an upward arrow indicates upward motion. Orange dots highlight areas where the difference between QBOE and QBOW is statistically significant at the 5% level using a one-tailed Student’s t-test.

To understand why the MJO-QBO relationship appears to have emerged only since the early 1980s, we first review why the MJO appears to be modulated by the QBO during the most recent period. Hendon and Abhik^31^ argue that the MJO is strengthened during QBOE because of a combination of reduced stability at the tropopause driven by the QBOE and by the convective phase of the MJO. Following a similar approach to Hendon and Abhik^31^, the vertical structure of the MJO during QBOE and QBOW is derived by regressing temperatures and zonal/vertical winds onto the RMM1 + RMM2 index, which represents MJO phases 5–6 (active MJO convection east of the Maritime Continent)^51^. Here we use the RMM1 + RMM2 index, OLR, temperatures and winds as calculated from JRA. Similar results are obtained if we use the WH RMM1 + RMM2 index and AVHRR OLR^31^. The lower panels of Fig. 4 display the longitude-vertical structure of the equatorial temperature and zonal and vertical wind anomalies when the convective phase of the MJO is crossing the Maritime Continent. The OLR anomaly, which is indicative of the convective anomaly, is displayed across the bottom of each panel. The positive tropospheric temperature anomaly to the east of the convective center is stronger, extends deeper into the upper troposphere on its western flank and is more in phase with the convective anomaly during QBOE as compared to QBOW. The overriding cold cap above the active convective anomaly at ~100 hPa is also stronger during QBOE. For both QBOE and QBOW the cold cap exhibits an eastward tilt with height into the lower stratosphere, indicative of a vertically propagating Kelvin wave with ~5 km wavelength, but the propagation extends further into the stratosphere during QBOE consistent with the Kelvin wave not being able to propagate upward in the presence of QBO westerlies. The enhanced warm anomaly and stronger cold cap act together to produce a stronger destabilization of the tropopause region that is more in phase with enhanced MJO convection than during QBOW. The key reason that the MJO is strengthened during QBOE is because the QBO-induced cold temperature anomaly extends down to ~100 hPa (the mean QBO temperature anomalies are contoured in Fig. 4), so that it constructively adds to the destabilization produced where MJO convection drives the cold cap anomaly. During QBOW, the QBO-induced warm anomalies at the tropopause act against the tropopause destabilization driven by the MJO. There is a much larger longitudinal-height area near the tropopause where the differences are significant between QBOW and QBOE in the more recent period (1980–2014) compared with the earlier period (1959–1979), when the behavior of the MJO showed little difference between QBO phases.

**Figure 5:**
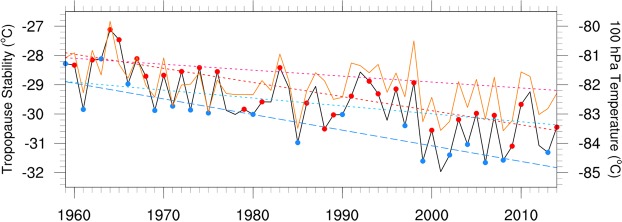
Time series of DJF warm-pool mean (10°S-10°N, 45°E-180°) temperature at 100 hPa (orange curve) and the difference between the temperature at 100 hPa and 200 hPa (black curve; tropopause stability) using JRA-55 reanalyses. The pink and red dotted lines represent linear trends computed during strong QBOW for 100 hPa temperature and tropopause stability, respectively, while the cyan and blue dotted lines represent linear trends computed during strong QBOE for 100 hPa temperature and tropopause stability, respectively. Blue/red dots indicate strong QBOE/QBOW years, respectively.

A probable mechanism for the emergence of the relationship between the QBO and the MJO is thus the recent trend in temperatures near the tropical tropopause layer (TTL). Figure 5 displays the time series of warm pool (10°S-10°N, 45°E-180°) mean temperature at 100 hPa and the difference in temperature at 100 and 200 hPa from the JRA-55 reanalysis for 1959–2014. The lower stratosphere has cooled and the upper troposphere has warmed, consistent with satellite and radiosonde observations^33,52,53^, thus acting to decrease the static stability at the TTL. Importantly, the temperature and stability trends computed separately in QBOE and QBOW years show similar negative trends, thus indicating that there is no apparent trend in the amplitude of the QBO in tropopause temperatures that might account for the strengthening relationship of the MJO with the QBO.

We note that the temperature trends from JRA-55 agree well with temperature trends from the European Centre for Medium-Range Weather Forecasts-Interim (ERA-Interim)^54^ reanalysis and the National Aeronautics and Space Administration Modern-Era Retrospective analysis for Research and Applications, Version 2 (NASA-MERRA2)^55^ reanalysis at 200 hPa (Supplementary Fig. 6) but disagree at 100 hPa (Supplementary Fig. 7). However, it was noted in Simmons *et al*.^56^ that ERA-Interim was cold-biased at 100 hPa compared with radiosondes. This cold bias was corrected in late 2006 when GPS radio occultation data began being assimilated into that reanalysis, and since that time, ERA-Interim has been in much closer agreement with JRA-55. We note that a similar close agreement between NASA-MERRA2 and JRA-55 at 100 hPa began around the same time.

**Figure 6:**
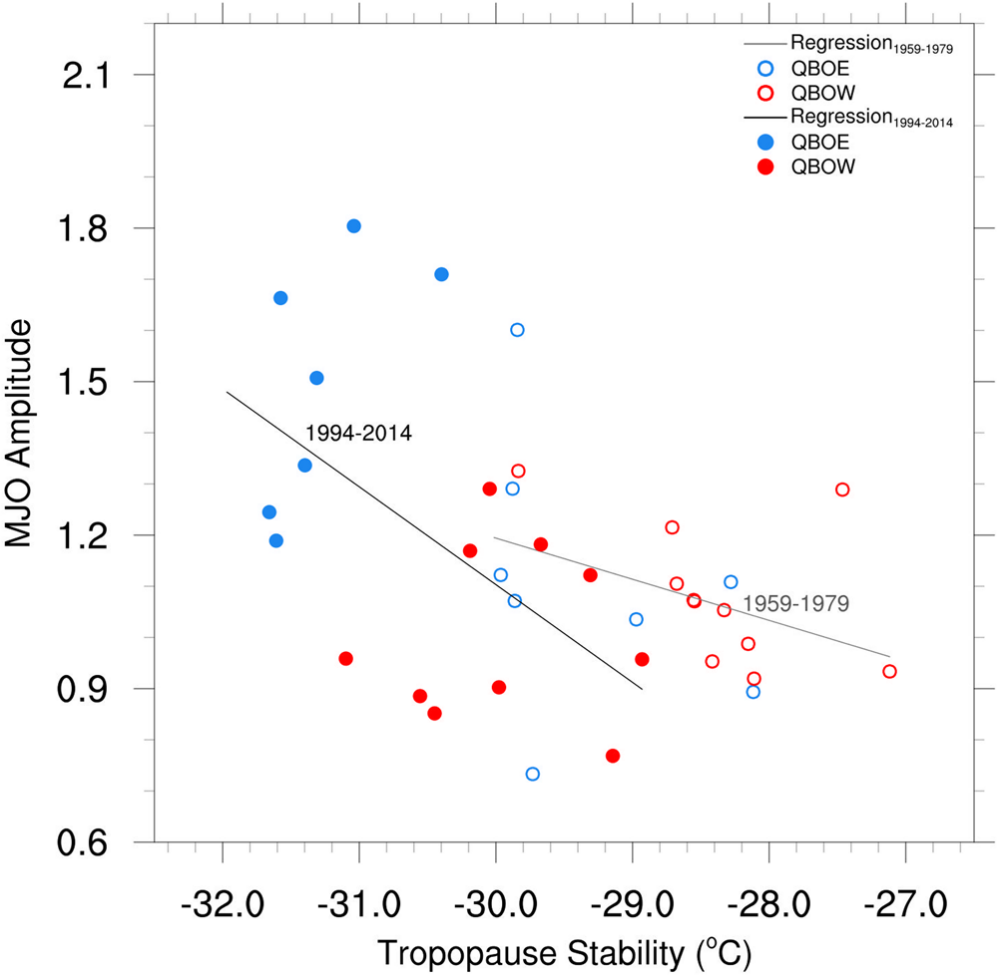
Scatterplot of DJF warm pool-averaged tropopause stability (100 minus 200 hPa T) (°C) versus DJF-averaged MJO amplitude for QBO east years (blue) and QBO west years (red) for the sub-periods from 1959–1979 (unfilled circles) and 1994–2014 (filled circles). Dashed lines represent the slope of the regression line for both sub-periods. The correlation between MJO amplitude and tropopause stability is −0.38 from 1959–1979 and −0.58 from 1994–2014.

The relationship between the seasonal mean stability at the TTL in the warm pool and mean amplitude of the MJO is displayed in Fig. 6. Reduced static stability is clearly associated with enhanced MJO amplitude, and the largest reduction of static stability and greatest MJO amplitude tend to occur during recent QBOE years. The linear relationship between static stability and MJO amplitude is markedly weaker during 1959–1979 than during the more recent period from 1994–2014. These results suggest that the recent temperature trends about the tropopause may have caused a threshold to have been reached such that MJO convection is now destabilized more during QBOE years than during QBOW years.

Consistent with this possibility of a threshold being crossed, there is also little apparent difference in magnitude and vertical structure of the MJO during QBOE and QBOW in the earlier 1959–1979 period (top panels Fig. 4). The aforementioned trends in near tropopause temperatures show that the QBO temperature anomaly during the earlier period (computed relative to the climatology for 1980–2014) is less cold during QBOE (upper left Fig. 4) and warmer during QBOW. Thus, there was less net destabilization at the tropopause in the vicinity of MJO convection during the earlier period.

Although Hendon and Abhik^31^ argue for a direct deepening of MJO convection by the reduced tropopause stability driven by the QBOE, the cooling trend in the lower stratosphere has also led to an increase in the formation of TTL ice clouds^4,5^, which might then be more strongly modulated by the MJO during QBOE. This MJO modulation, whereby TTL cirrus is enhanced in the cold cap driven by the MJO^51^, could act to radiatively destabilize the tropospheric column in phase with MJO convection, thus contributing to deeper MJO convection during QBOE. This is an area for further investigation.

We have documented that a robust relationship between the QBO and amplitude of the MJO during the boreal winter has only emerged since the early 1980s and appears driven by the recent trend in temperatures about the TTL. These temperature trends appear to be a combination of anthropogenic climate change (e.g., ozone depletion and increasing greenhouses gases)^53^ and natural variability of tropical ocean surface temperature^33^. Consequently, we may expect that the relationship between the QBO and MJO amplitude could continue to change in the future. If the recent tropopause temperature trends persist, we might anticipate MJO activity during QBOW to soon begin to increase because the warm anomalies at 100 hPa during QBOW are currently just warmer than the cold anomalies during QBOE that were occurring in the 1970s before the strong MJO-QBO relationship began to emerge.

## Supplementary information

Supplementary Information for

## Data Availability

All data generated and analyzed during the course of this study is available from the corresponding author upon request.
