# Supplementary material for: On the emerging relationship between the stratospheric Quasi-Biennial oscillation and the Madden-Julian oscillation
Source: Sci Rep. 2019 Feb 27;9:2981. doi: 10.1038/s41598-019-40034-6 (PMC6393487; doi:10.1038/s41598-019-40034-6)
Supplement: Supplementary file 1 — Supplementary Information for [file 41598_2019_40034_MOESM1_ESM.pdf]

Supplementary Information for:

On the emerging relationship between the stratospheric Quasi-Biennial oscillation and the Madden-Julian oscillation

Authors: P. Klotzbach[1]*, S. Abhik[2,3], H. H. Hendon[3], M. Bell[1], C. Lucas[3], A. Marshall[4], E. C. J. Oliver[5]

26 January, 2019

Affiliations:

[1]Department of Atmospheric Science, Colorado State University, Fort Collins, CO, USA.

[2]School of Earth, Atmosphere & Environment, Monash University, Clayton, Australia.

[3]Bureau of Meteorology, Melbourne, Australia.

[4]Bureau of Meteorology, Hobart, Australia.

[5]Department of Oceanography, Dalhousie University, Halifax, Nova Scotia, Canada.

*Corresponding author. E-mail: philk@atmos.colostate.edu

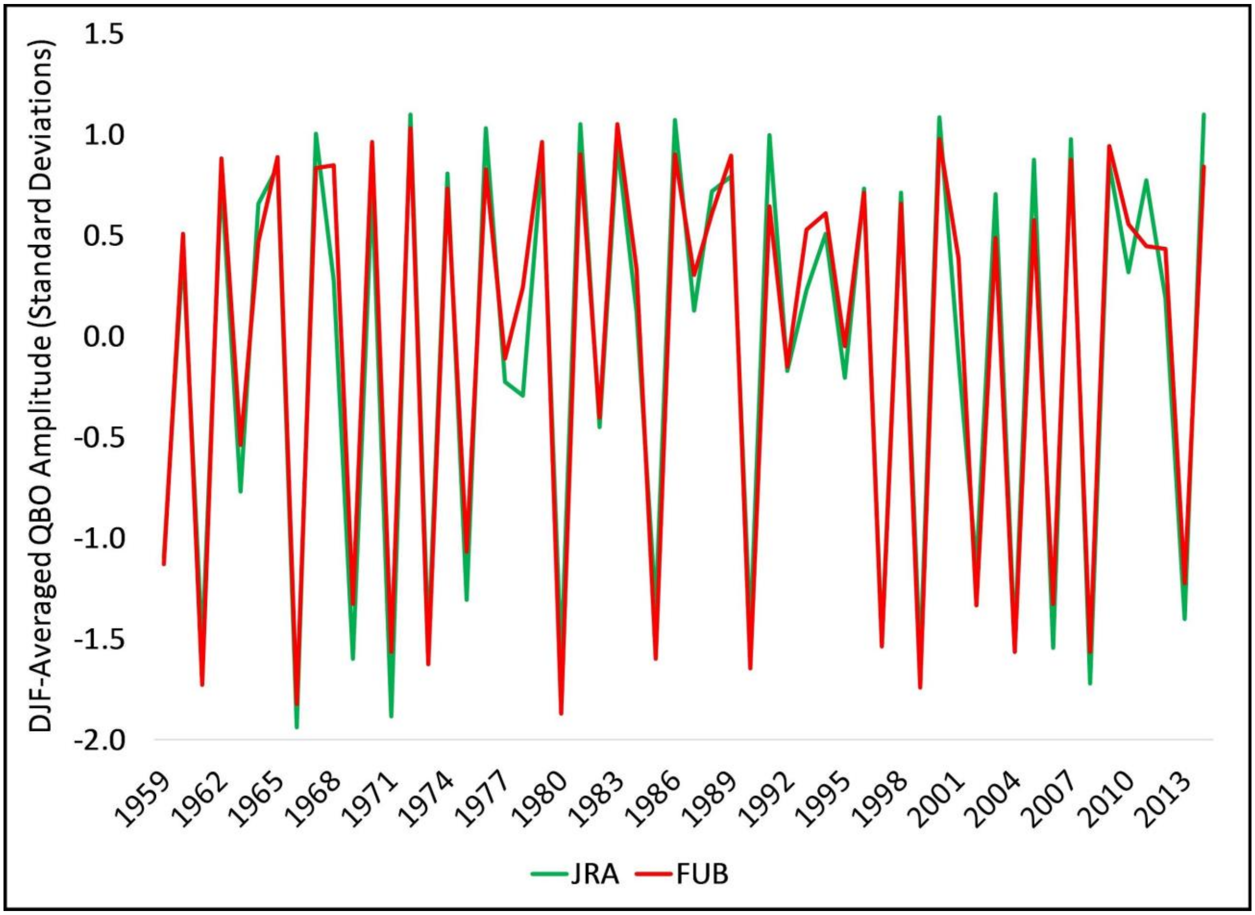

Fig. S1. Time series of the standardized U50 QBO index from FUB (red curve) and JRA-55 (green curve). The correlation between the two timeseries is 0.98.

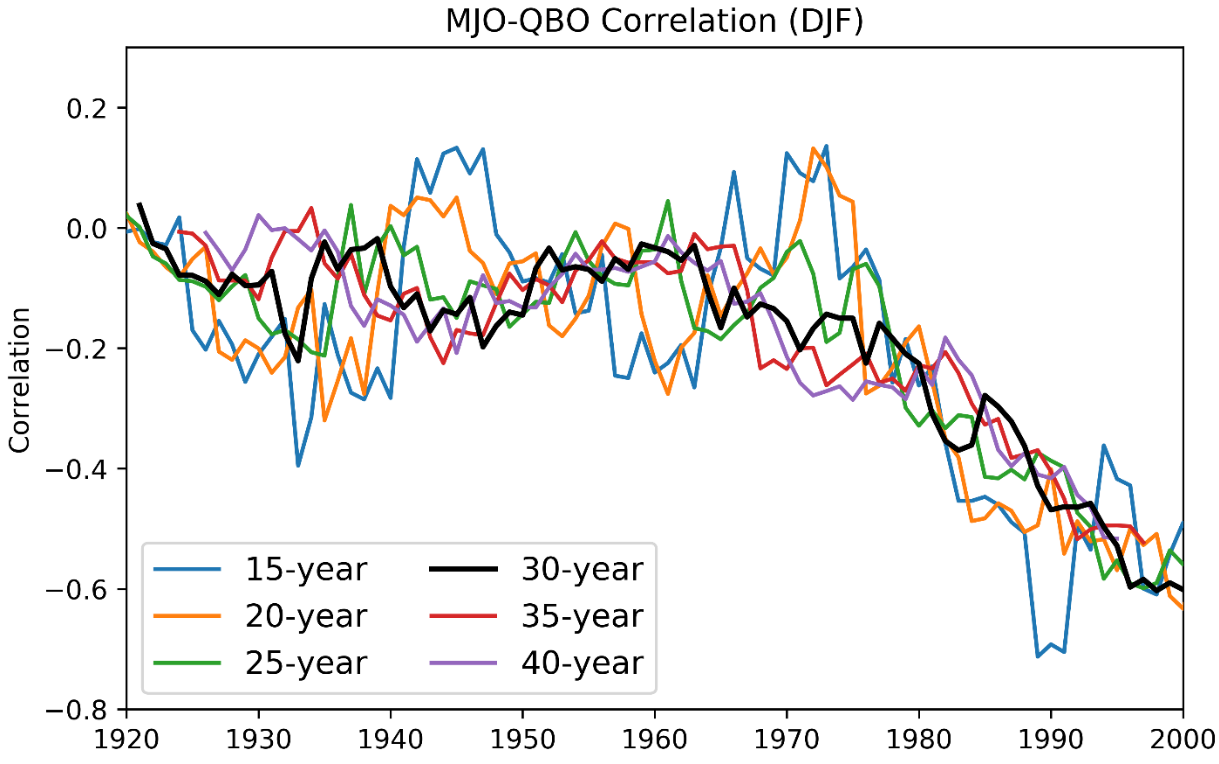

Fig. S2. DJF QBO-MJO correlation magnitude using different correlation lengths between 15 and 40 years. The ordinate on the x-axis is given by the central year of the running window.

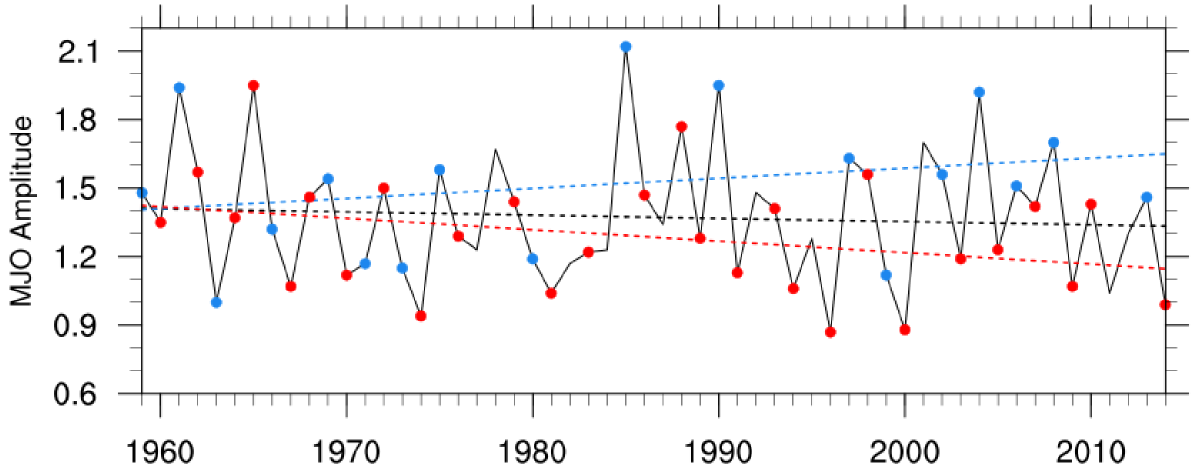

Fig. S3. Time series of DJF MJO amplitude from OT. Trends are displayed during east QBO years (blue dotted line), west QBO years (red dotted line) and all years (black dotted line). The blue and red circles represent strong east and west QBO years, respectively.

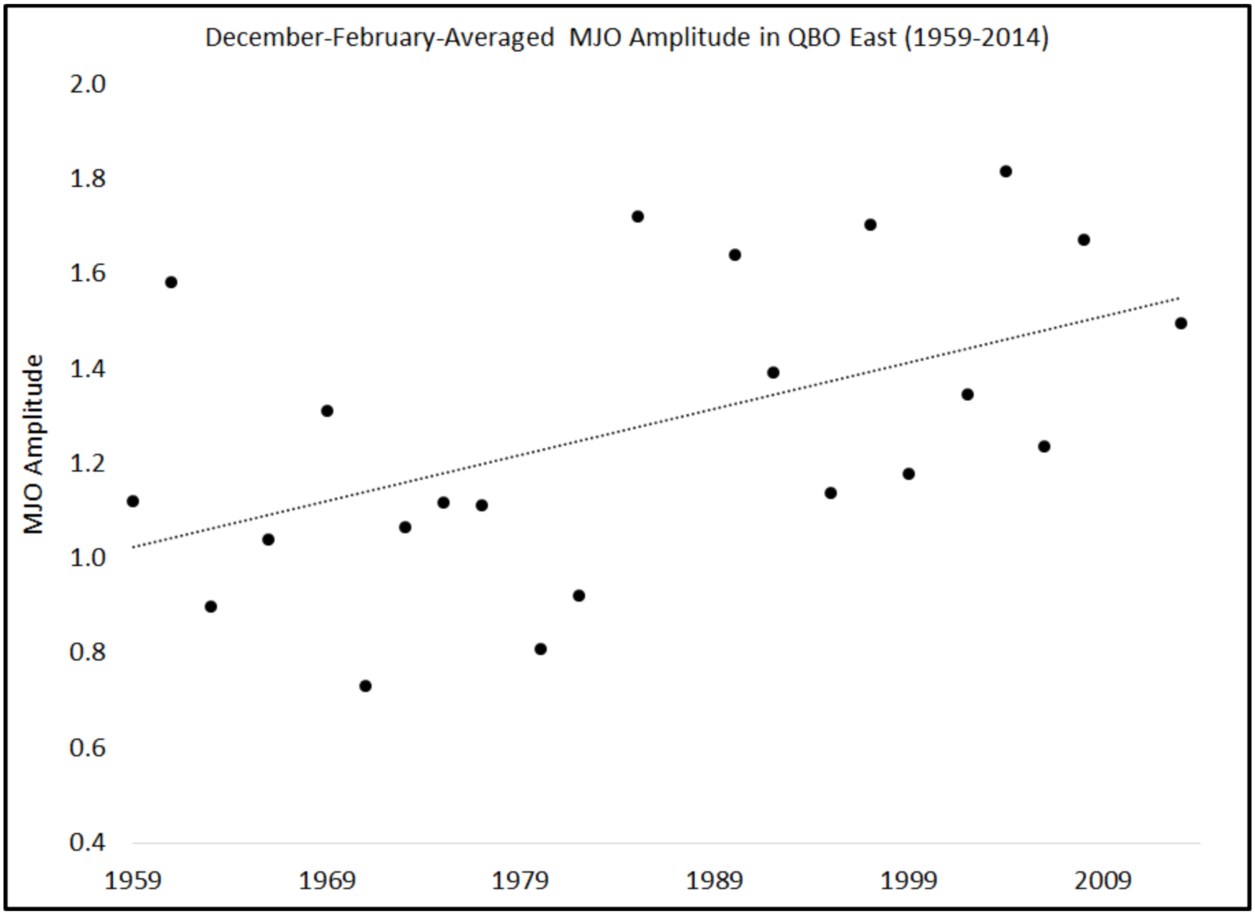

Fig. S4. DJF-averaged MJO amplitude in QBOE from 1959-2014.

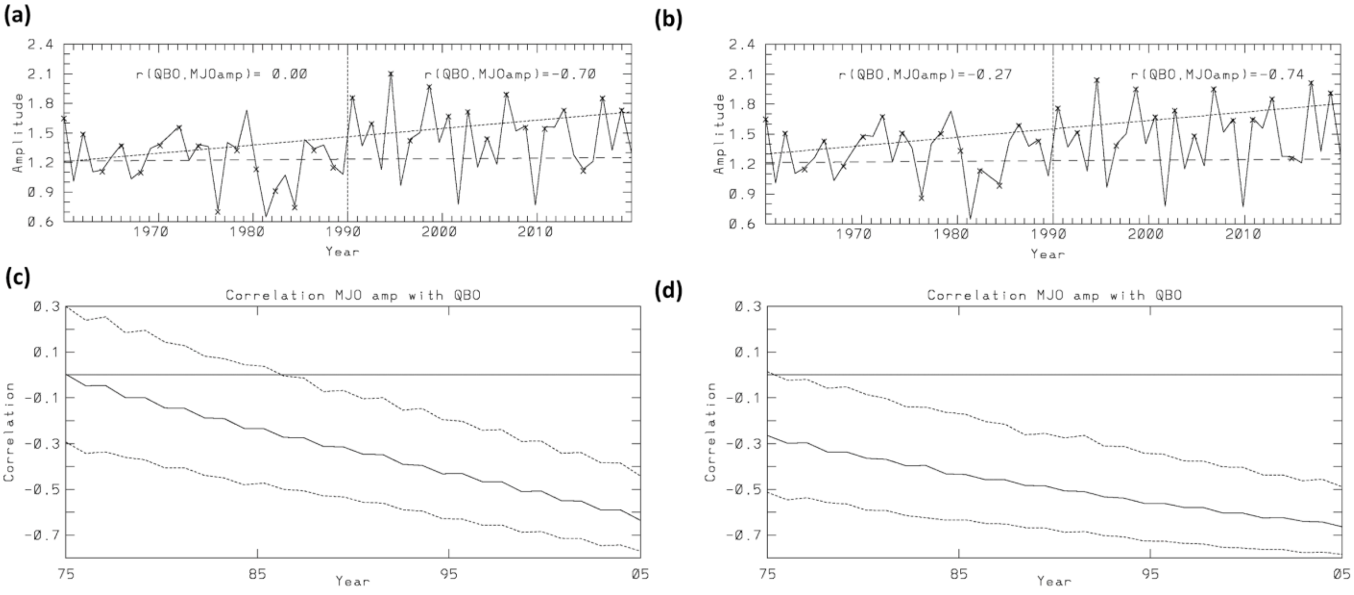

Fig. S5. (panel a) Synthetic time series of MJO amplitude given by MJOA(t)=MJOA(0)+trend(t)+noise(t) during QBOE and MJOA(t)=MJOA(0)+noise(t) during QBOW. The QBO is assumed to oscillate with a 2-year periodicity. The initial MJO amplitude MJOA(0) = 1.2 amplitude units. The trend in QBOE is prescribed trend(t)= t*scale, with scale = 0.01 amplitude units per year. The noise is prescribed to be Gaussian white noise with standard deviation = 0.25 amplitude units. Over the 60-year period, the net trend is ~2 times the standard deviation of the noise, which matches the observed behavior using the JRA index in Fig. 3. The year labels on the abscissa are arbitrary. QBOE years are indicated by asterisks. The thin dotted line is the trend in the MJO amplitude computed during QBOE. The long dashed line is the trend in MJO amplitude computed during QBOW. For this example the correlation of the synthetic QBO index with synthetic MJO amplitude in the first and second thirty years is -0.27 and -0.74, respectively. Using these settings for the standard deviation of the noise and the magnitude of the trend in MJO amplitude during QBOE, 1000 samples of the 60-year time series were created. (panel b) As in panel a except MJO amplitude is given by MJOA(t)=MJOA(0)+ noise(t) during

both QBOW and QBOE during the first 30 years, while in the second 30 years MJOA(t)=MJOA(0)+ noise(t) during QBOW and MJOA(t)=MJOA(0)+ noise(t)+ constant during QBOE. The noise settings are as in panel a and the constant increase in MJO amplitude during QBOE during the second 30 years is a constant = 0.03 amplitude units. This constant increase yields a similar overall trend in amplitude during QBOE as in the first example and also matches the observed "step change" in MJO amplitude. Using these settings for a step change in MJO amplitude in the second 30 years during QBOE, 1000 samples of the 60-year time series were created. (panel c) Median (solid) and 5th and 95th percentile (dotted) correlations between the QBO index and the MJO amplitude computed in 30 year sliding windows based on the 1000 samples of synthetic data used to plot panel a. (panel d) As in panel c but for the 1000 samples of synthetic data used to plot panel b.

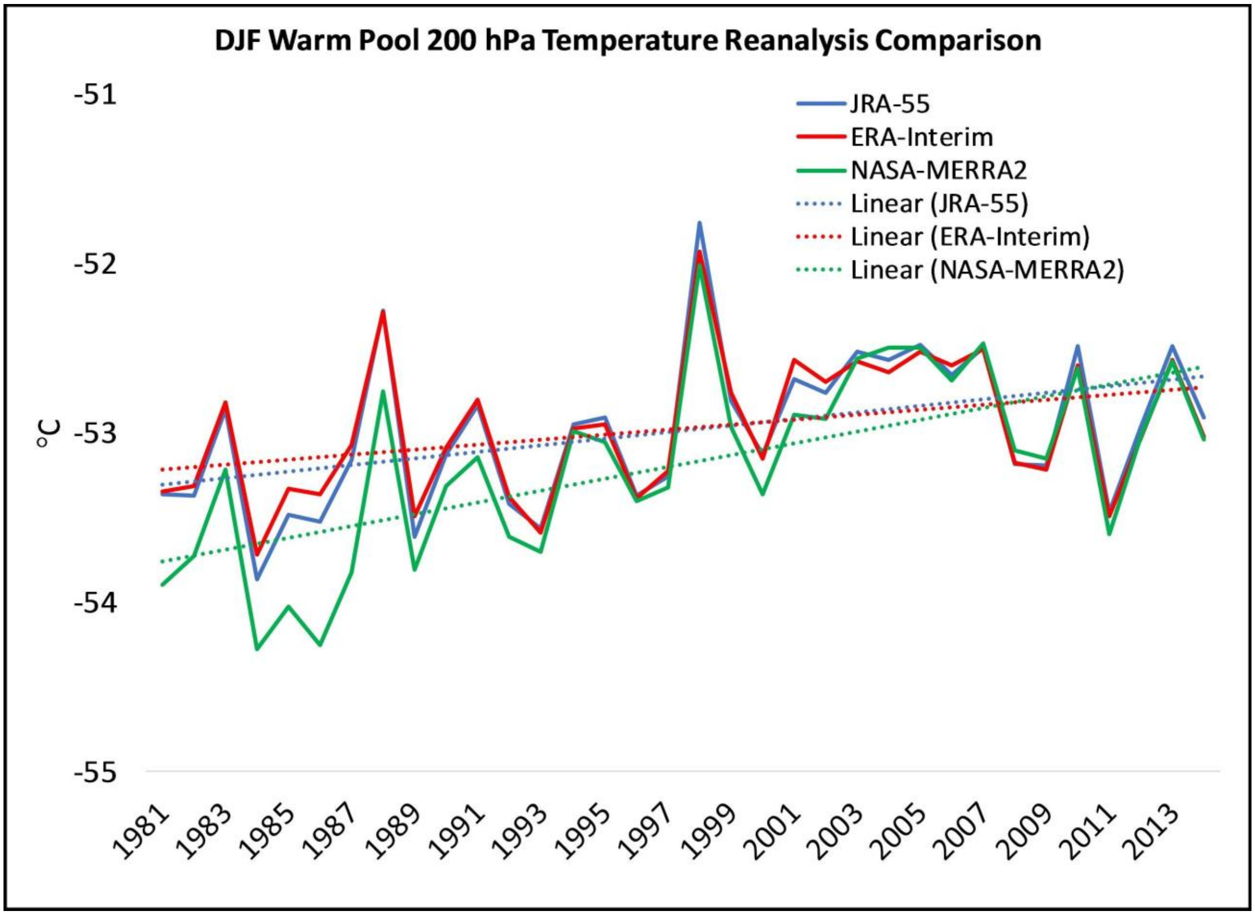

Fig. S6. Time series of DJF 200 hPa warm pool temperature from three different reanalysis products: JRA-55 (blue line), ERA-Interim (red line) and NASA-MERRA2 (green line). Linear trends (dashed lines) for each timeseries over the period from 1981-2014 are also plotted.

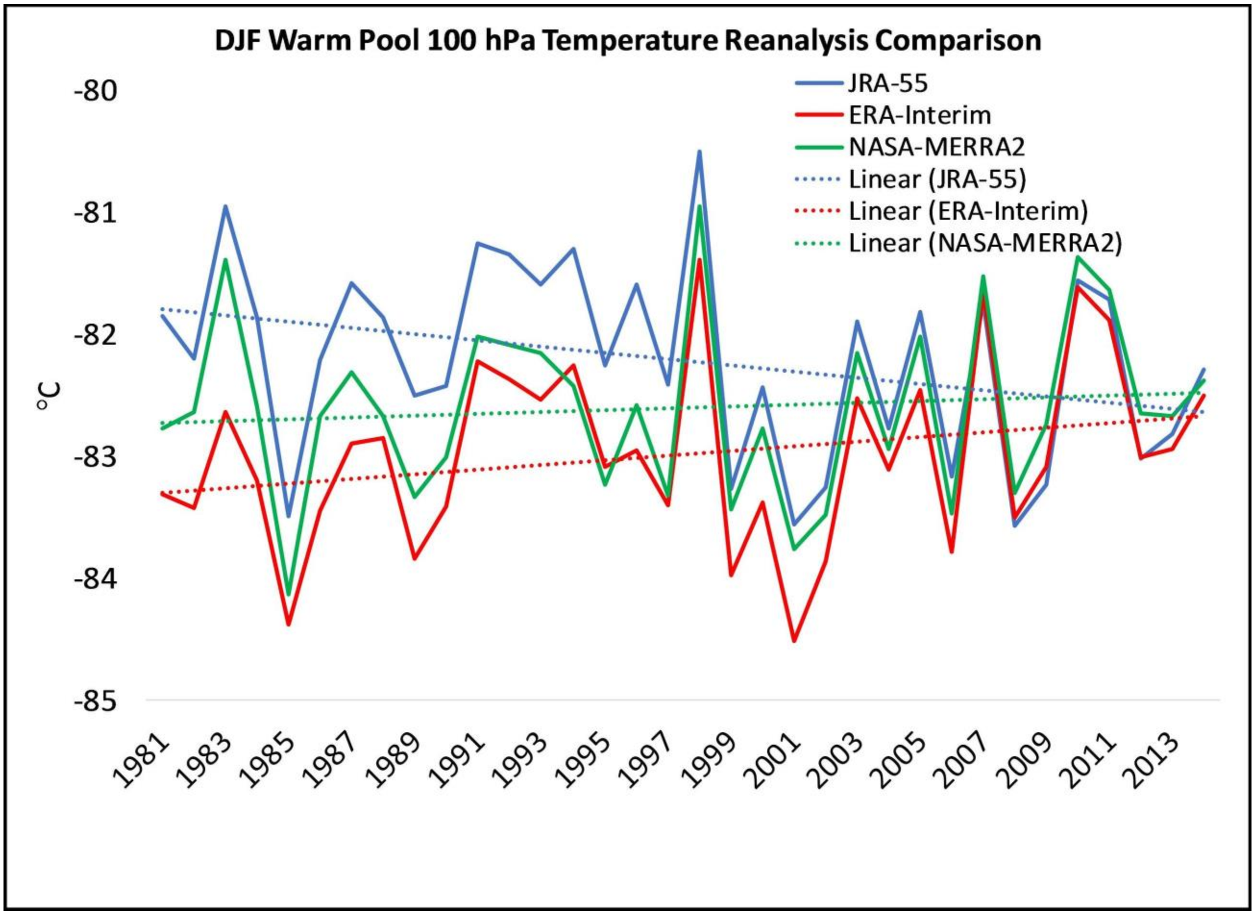

Fig. S7. Time series of DJF 100 hPa warm pool temperature from three different reanalysis products: JRA-55 (blue line), ERA-Interim (red line) and NASA-MERRA2 (green line). Linear trends (dashed lines) for each timeseries over the period from 1981-2014 are also plotted.
